# Expression and Regulation of Cyclic Nucleotide Phosphodiesterases in Human and Rat Pancreatic Islets

**DOI:** 10.1371/journal.pone.0014191

**Published:** 2010-12-01

**Authors:** Emilia Heimann, Helena A. Jones, Svante Resjö, Vincent C. Manganiello, Lena Stenson, Eva Degerman

**Affiliations:** 1 Department of Experimental Medical Science, Division for Diabetes, Metabolism and Endocrinology, Lund University, Lund, Sweden; 2 Pulmonary/Critical Care Medicine Branch, National Heart, Lung, and Blood Institute, National Institutes of Health (NIH), Bethesda, Maryland, United States of America; University of Bremen, Germany

## Abstract

As shown by transgenic mouse models and by using phosphodiesterase 3 (PDE3) inhibitors, PDE3B has an important role in the regulation of insulin secretion in pancreatic β-cells. However, very little is known about the regulation of the enzyme. Here, we show that PDE3B is activated in response to high glucose, insulin and cAMP elevation in rat pancreatic islets and INS-1 (832/13) cells. Activation by glucose was not affected by the presence of diazoxide. PDE3B activation was coupled to an increase as well as a decrease in total phosphorylation of the enzyme. In addition to PDE3B, several other PDEs were detected in human pancreatic islets: PDE1, PDE3, PDE4C, PDE7A, PDE8A and PDE10A. We conclude that PDE3B is activated in response to agents relevant for β-cell function and that activation is linked to increased as well as decreased phosphorylation of the enzyme. Moreover, we conclude that several PDEs are present in human pancreatic islets.

## Introduction

Cyclic nucleotide phosphodiesterases (PDEs) are enzymes with the function to hydrolyze cyclic AMP (cAMP) and cyclic GMP (cGMP) [1], [2]. There are eleven known PDE families (PDE1-11) with a total of 21 gene products and 100 resulting mRNA products [1], [2]. The PDE families differ in primary structures, affinities for cAMP and cGMP, responses to specific effectors, sensitivities to specific inhibitors, mechanisms whereby they are regulated, cellular expression and intracellular location [1], [2]. Indeed, it is believed that individual isozymes modulate distinct regulatory pathways within the cell [1], [2]. Family-selective PDE inhibitors available for several PDEs have been very useful in dissecting out specific functions for selected PDEs and are also used in the clinic, as well as being developed for the treatment of various diseases [1], [2].

It is well established that PDE1, PDE3, and PDE4 are expressed in rodent pancreatic islets and β-cells [3], [4], [5], [6], [7], [8], [9], [10]. Furthermore, several studies have shown that family-selective inhibition of PDE1, PDE3 and to some extent also PDE4 potentiates glucose-stimulated insulin secretion (GSIS) [3], [4], [10], [11], [12]. More recently mRNAs for PDE1B-C, PDE2A, PDE3A-B, PDE4A-D, PDE5A, PDE8A-B, PDE9A, PDE10A and PDE11A as well as the proteins PDE3A-B, PDE4B and PDE8A have been detected in rodent pancreatic islets and β-cell lines [8], [10], [11], [13]. Of these PDEs, PDE8B and PDE10A have potential in the context of β-cell function, since diminished activity of PDE8B [13] as well as PDE10A inhibition [14] potentiated insulin secretion in response to glucose in rat pancreatic islets.

With regard to PDE3B, its physiological and functional role has been extensively studied in pancreatic β-cells *in vivo* and *in vitro* [7], [8], [11], [15]. It has been shown that β-cell PDE3B is localized to the insulin granules and the plasma membrane, where it appears to regulate the acute first phase and the second sustained phase of insulin secretion [8]. Further, RIP-PDE3B mice overexpressing PDE3B specifically in β-cells show impaired GSIS as well as cAMP-potentiated GSIS, impaired glucose tolerance and increased sensitivity to high-fat induced insulin resistance [7], [8], [11], [15]. Thus, it appears that PDE3B has an important role in pancreatic β-cells with regard to the regulation of insulin secretion and also the regulation of whole body energy homeostasis in mice. However, very little is known about the regulation of PDE3B activity in β-cells. Also, the information is sparse with regard to the expression and activity pattern of PDEs in human pancreatic islets. To our knowledge one study, however, indicates the presence of PDE3 and PDE4 activities as well as modest activity of PDE1 in human islets, and inhibition of PDE3, but not PDE1 and PDE4, was shown to increase insulin secretion [9].

The aim of this work was to study (a) the alterations in PDE3B activity and phosphorylation state in response to agents of relevance for insulin secretion as well as (b) the expression and activity of selected PDEs in human pancreatic islets. We show that glucose and insulin, as well as forskolin, a cAMP-elevating agent, activate PDE3B in rat pancreatic islets and/or INS-1 (832/13) cells. The activation was associated with altered phosphorylation states of the enzyme. We also show that PDE1, PDE3, PDE4C, PDE7A, PDE8A and PDE10A are expressed in human pancreatic islets.

## Materials and Methods

### 2.1 Animal Model

Sprague Dawley rats were purchased from Charles River Laboratories (Germany) and kept under standardized conditions in the animal house facilities. All experimental procedures have been approved by the Committee of ethical animal research in Malmö and Lund (permission number: M166-08).

### 2.2 Cell Culture

The rat insulinoma cell line INS-1 (832/13) [a modified INS-1 cell clone, stably transfected with the human proinsulin gene) [16] (passages 70–90)], was kept in RPMI 1640 (Sigma), containing 11 mM glucose and supplemented with 10% fetal calf serum, 100 units/ml penicillin, 100 µg/ml streptomycin, and 50 µM β-mercaptoethanol. The cells were grown at 37°C in an atmosphere of 5% CO_2_ and 95% air.

### 2.3 Isolation of Pancreatic Rat Islets

Pancreatic islets from 5–6 weeks old male Sprague Dawley rats were isolated by a collagenase digestion technique [15]. In short, the common bile duct was cannulated and ligated at the Papilla Vateri. The pancreas was filled with 10 ml of ice-cold Hank's balanced salt solution (HBSS) (Sigma-Aldrich) supplemented with 1.3 units/ml Collagenase P (Roche), removed and then incubated at 37°C for 23 minutes. After a few washes in HBSS, pancreatic islets were collected under a stereomicroscope and incubated at 37°C overnight in RPMI 1640, supplemented with 5 mM glucose (Invitrogen).

### 2.4 Treatment of Cells and Rat Pancreatic islets

Rat pancreatic islets or INS-1 (832/13) cells were pre-incubated for 2 hours in Krebs-Ringer bicarbonate buffer (KRBB) containing 1 mM glucose, 10 mM Hepes, pH 7.2–7.4, 120 mM NaCl, 5 mM NaHCO_3_, 5 mM KCl, 1.2 mM KH_2_PO_4_, 2.5 mM CaCl_2_, 1.2 mM MgSO_4_, and 0.2% BSA. The buffer was changed and islets (80–140 islets per stimulus) or cells were stimulated with KRBB supplemented with 16 mM glucose, 100 nM insulin or 100 µM forskolin. In some experiments, islets were first incubated in 250 µM diazoxide (in KRBB supplemented with 1 mM glucose) for 30 minutes and then stimulated with 16 mM glucose. For K^+^-stimulation the buffer was changed to KRBB, containing 10 mM Hepes, pH 7.2–7.4, 60 mM NaCl, 5 mM NaHCO_3_, 60 mM KCl, 1.2 mM KH_2_PO_4_, 2.5 mM CaCl_2_, 1.2 mM MgSO_4_, and 0.2% BSA, supplemented with 1 mM glucose. After 1 hour stimulation, islets or cells were harvested in a buffer containing 50 mM TES, pH 7.4, 250 mM sucrose, 1 mM EDTA, 2 mM EGTA, 40 mM phenyl-phosphate and 5 mM NaF supplemented with Complete Protease Inhibitor Cocktail (containing inhibitors for serine-, cysteine- and metalloproteases as well as calpains) (Roche) and homogenized by 10 short sonication pulses. The homogenates were briefly centrifuged to remove cell debris. Total protein amount was determined according to Bradford [17] and PDE3 activity was measured (15 islets per assay tube) as described below.

### 2.5 Treatment of Human Pancreatic islets

Human islets from non-diabetic individuals were provided by the Nordic Network for Clinical Islet Transplantation (O. Korsgren, Uppsala University, Sweden) and characterized by the Human Tissue Laboratory (Jalal Taneera, Lund University Diabetes Centre, Sweden). All experimental procedures have been approved by the Regional ethical committee in Lund (permission number: 173/2007). Frozen human pancreatic islets were thawed, resuspended in a buffer containing 50 mM TES, pH 7.4, 250 mM sucrose, 1 mM EDTA, and 0.1 mM EGTA, supplemented with Complete Protease Inhibitor Cocktail (Roche) and homogenized by 10 short sonication pulses. Total protein amount was determined according to Bradford [17].

### 2.6 PDE Assay

PDE activity was measured in duplicates as described [18] in the presence or absence of family-selective PDE inhibitors. The following PDE inhibitors were used: 3 µM of the PDE3 inhibitor OPC3911 (Osaka Inc. Japan), 10 µM of the PDE4 inhibitor RO-20-1724 (Roche) or 50 µM of the PDE1 inhibitor 8MM-IBMX (Biomol). Assays were performed at 30°C in a total volume of 300 µl of buffer containing 50 mM TES pH 7.4, 250 mM sucrose, 1 mM EDTA, 0.1 mM EGTA, and 8.3 mM MgCl_2_, 0.5 µM cAMP, 0.5 µg ovalbumin and 1 µCi/ml [3H] cAMP.

### 2.7 Adenovirus Infection

To overexpress PDE3B INS-1 (832/13) cells were infected with an adenovirus expressing flag-tagged mouse PDE3B (AdPDE3B) [11] or a control virus expressing β-galactosidase (Adβ-gal). High titer viral stocks (∼10^10^ pfu/ml) were used to infect cells kept in RPMI 1640 (Invitrogen), containing 11 mM glucose, for 2 hours. For experiments, conducted 18 hours post infection, cells were treated as described above.

### 2.8 ^32^P labelling and Treatment of INS-1 (832/13) Cells

AdPDE3B-infected INS-1 (832/13) cells (see above) were washed in low phosphate KRBB (LP-KRBB) (10 mM Hepes, pH 7.2–7.4, 120 mM NaCl, 5 mM NaHCO_3_, 5 mM KCl, 50 µM KH_2_PO_4_, 2.5 mM CaCl_2_, 1.2 mM MgSO_4_, and 0.2% BSA) and pre-incubated in LP-KRBB containing 1 mM glucose and ^32^P at 0.2 mCi/ml for 2 hours. The cells were stimulated at 37°C for 1 hour and homogenized (sonication, 10 pulses) on ice in 50 mM TES, pH 7.4, 250 mM sucrose, 1 mM EDTA, 2 mM EGTA, 40 mM phenyl-phosphate, 5 mM NaF, 1 mM DTE, 50 µM vanadate, 1 mM PMSF, 10 µg/ml leupeptin, 10 µg/ml antipain and 1 µg/ml pepstatin A. The homogenate was centrifuged at 175,000× g at 4°C for 1 h and the crude membrane fraction was resuspended and homogenized in the homogenization buffer described above, supplemented with 1% C_13_E_12_ (non-ionic alkyl polyoxyethylene glycol detergent from Berol Kemi AB, Stenungsund, Sweden).

### 2.9 Subcellular Fractionation

Cells were washed three times with chilled homogenization buffer containing 250 mM sucrose, 0.5 mM EGTA, 5 mM HEPES adjusted with KOH to pH 7.4, supplemented with 10 µg/ml leupeptin, 10 µg/ml antipain and 1 µg/ml pepstatin (Peptide Institute Inc., Osaka, Japan), 5 mM NaF, 1 mM DTE, 50 µM vanadate and 1 mM PMSF. Cells were harvested in homogenization buffer and disrupted under 350 psi of nitrogen in a Parr bomb (Parr Instrument Company, Illinois, USA) for 15 min at 4°C. A fraction of the homogenate was centrifuged at 700× g for 15 min at 4°C and the resulting supernatant was mixed with sucrose (final concentration 250 mM) and Percoll (15% of final mixture). A self-generating gradient was produced through centrifugation at 48,000× g for 25 min in a fixed angle rotor. The plasma membrane (top) and insulin granule (bottom) fractions were collected and washed three times in 4 volumes of homogenization buffer through 150,000× g centrifugation at 4°C for 30 min. The cytosol fraction was obtained by centrifugation of the homogenate at 150,000× g at 4°C for 60 min.

### 2.10 SDS-PAGE and Immunoblot Analysis

Homogenates from cells, islets or immuno-precipitates were subjected to SDS-PAGE. Proteins were electrotransferred to polyvinylidene membranes (Millipore) and the membranes were stained with Ponceau S (0.1% in 5% acetic acid) and then blocked with 5% milk in a buffer consisting of 20 mM Tris-HCl, pH 7.6, 137 mM NaCl and 0.1% (v/w) Tween-20 for 30–60 min. Membranes with proteins from cells or immuno-precipitates were probed with PDE3B antibodies (prepared against the peptide CGYYGSGKMFRRPSLP from rat PDE3B sequence [19]), or mouse monoclonal anti-Na^+^/K^+^-ATPase antibodies, for 16 h. Membranes with proteins from human islets were probed with the following antibodies: PDE4C, PDE7A, PDE8A or PDE10A (Scottish Biomedical), and incubated overnight. Proteins were detected using the chemiluminescent Super Signal West Pico Luminol/Enhancer solution from Pierce (Illinois, USA) and a Fuji LAS 1000 Plus system or a Fuji LAS 3000 Plus system (Fuji Photo Film Co., Ltd, Tokyo, Japan).

### 2.11 Statistics

Data are presented as means ± SEM from the indicated number of experiments. Statistically significant differences were analyzed using Wilcoxon's Signed Rank test or Student's t-test with significance levels *p<0.05, **p<0.01 and ***p<0.001.

## Results

### 3.1 Activation of PDE3B in rat pancreatic islets and INS-1 (832/13) cells

Glucose-stimulated insulin secretion (GSIS) involves metabolization of glucose, which generates an increase in intracellular ATP followed by closing of the ATP sensitive K_ATP_ channels [20]. This closure results in membrane depolarization, opening of voltage-dependent Ca^2+^ channels and an influx of Ca^2+^, which triggers exocytosis of insulin granules [20]. GSIS is potentiated by hormones that increase cAMP, such as the incretins glucagon like peptide-1 (GLP-1) and glucose-dependent insulinotropic polypeptide (GIP) [21], [22], [23]. Also, insulin has been shown to induce inhibition as well as stimulation of GSIS [24].

**Figure 1:**
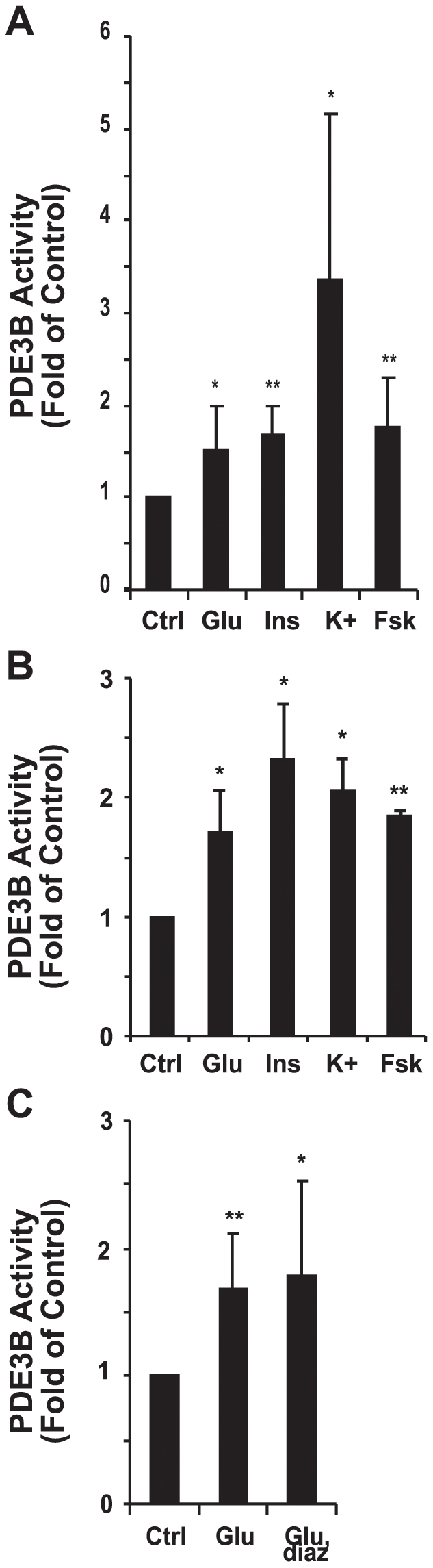
Activation of PDE3B in rat pancreatic islets and INS-1 (832/13) cells. Isolated rat pancreatic islets (A) or cells (B) were pre-incubated in low glucose for 2 hours and then stimulated with 16 mM glucose (glu), 100 nM insulin (ins), 60 mM K^+^ or 100 µM forskolin (fsk) for 1 hour. Isolated pancreatic islets (C) were pre-incubated in low glucose for 2 hours, pre-incubated in the presence or absence of 250 µM diazoxide (diaz) for 30 minutes and then stimulated with 16 mM glucose for 1 hour. Pancreatic islets or cells were homogenized and PDE3 activity was measured (n = 5–7 for A, n = 4 for B and n = 10 for C).

Activation of PDE3B was studied in rat pancreatic islets and rat derived INS-1 (832/13) cells. As shown in Fig. 1, high glucose (16 mM for 1 h), the main trigger of insulin secretion, resulted in activation of PDE3 in rat islets (Fig. 1A) and INS-1 (832/13) cells (Fig. 1B). Since insulin is a well known activator of PDE3B in adipocytes and hepatocytes [25], [26] we wanted to test the possibility that the insulin released from islets or INS-1 (832/13) cells could activate β-cell PDE3B and thus mediate the glucose-induced activation of PDE3B. As seen in Fig. 1, indeed, exogenously added insulin induced activation of PDE3B in islets (Fig. 1A) and INS-1 (832/13) cells (Fig. 1B). To elucidate if the glucose effect on PDE3B was dependent on the release of endogenously produced insulin, islets were treated with high glucose in the presence or absence of diazoxide. Diazoxide is a K_ATP_ channel-activator that hyperpolarizes the β-cell and prevents insulin secretion even in the presence of glucose. As shown in Fig. 1C, diazoxide did not inhibit glucose-mediated activation of PDE3B, indicating that glucose is not mediating its effect via endogenously produced insulin.

As a next step, we tested the possibility that depolarization of the β-cell mediated independently of glucose would activate PDE3B. For that purpose, cells and islets were stimulated with high (60 mM) K^+^ under non- stimulatory glucose conditions (1 mM), the rational for which is that elevated K^+^ concentration depolarizes the β-cell and directly triggers insulin secretion, thus bypassing glucose metabolism. As shown in Fig. 1, high K^+^ induced activation of PDE3B, indicating that metabolization of glucose is not necessary for activation of the enzyme. Islets and cells were also treated with forskolin, an agent known to activate adenylyl cyclases, leading to increased intracellular cAMP. As shown in Fig. 1, stimulation of rat pancreatic islets (Fig. 1A) and INS-1 (832/13) cells (Fig. 1B) with forskolin resulted in activation of PDE3B.

### 3.2 Phosphorylation of PDE3B in response to glucose, insulin and forskolin

**Figure 2:**
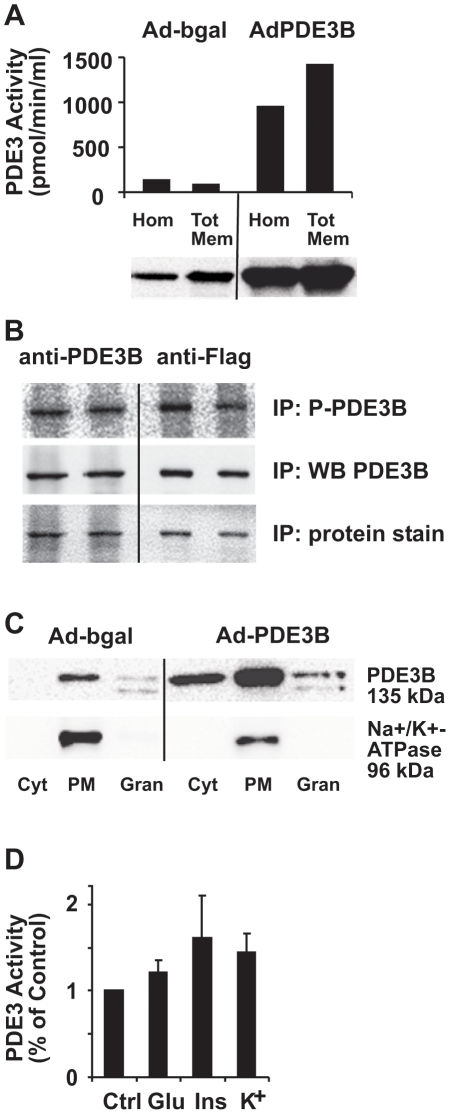
Characterization of AdPDE3B-overexpressed PDE3B in INS-1 (832/13) cells. A. INS-1 (832/13) cells were infected with either Ad-βgal or AdPDE3B virus for 2 hours, 16 hours prior to experiment. Cells were homogenized and analyzed by PDE3 activity assay or immunoblot analysis using anti-PDE3B antibody. B. Immunoisolation of recombinant ^32^P-labelled PDE3B from AdPDE3B infected INS-1 (832/13) cells. A crude membrane fraction was used as starting material for immunoprecipitation using anti-PDE3B antibody or anti-flag M2 gel. Thoroughly washed immunoprecipitates were run on SDS-PAGE and transferred to nitrocellulose membranes. Membranes were subjected to autoradiography, immunoblot analysis using anti-PDE3B antibodies and Ponceau S staining, respectively. C. Adβ-gal or AdPDE3B-infected INS-1 (832/13) cells were subjected to subcellular fractionation using density gradient centrifugation (Percoll™). Cytosol (Cyt), granule (Gran) and plasma membrane (PM) fractionations were prepared and analyzed with immunoblot analysis for PDE3B expression. The purity of the fractions was evaluated by immunoblot analysis of the plasma membrane marker Na^+^/K^+^-ATPase (n = 2). D. Cells were AdPDE3B-infected, pre-incubated in low glucose for 2 hours and then stimulated with 16 mM glucose, 100 nM insulin or 60 mM K^+^ for 1 hour. Cells were homogenized and PDE3 activity was measured (n = 3).

Initial experiments showed that in ^32^P-labelled INS-1 (832/13) cells, endogenous PDE3B was not present in sufficient amounts to be detected as a ^32^P-phosphorylated protein. Thus, to be able to study phosphorylation of PDE3B in β-cells we overexpressed PDE3B using an adenoviral system. The recombinant enzyme (AdPDE3B) was characterized with regard to expression level, localization and ability to be activated. We found that infection of INS-1 (832/13) cells with AdPDE3B virus resulted in a ∼10-fold overexpression of PDE3B compared to control Adβ-gal infected cells (Fig. 2A) and that ^32^P-PDE3B was detected after immunoprecipitation using both PDE3B antibody and an anti-flag M2 affinity gel (Fig. 2B), as well as using mass spectrometry (data not shown). To establish the intracellular localization of overexpressed PDE3B, INS-1 (832/13) cells expressing recombinant PDE3B were subjected to subcellular fractionation and subsequent immunoblotting. Fig. 2C demonstrates that, similar to endogenous PDE3B [8], recombinant PDE3B localizes to the granule and plasma membrane fractions, respectively. There is also a clear presence of recombinant PDE3B in the cytosol fraction not seen in control infected cells. A likely explanation could be that the membranes are saturated with PDE3B proteins as a consequence of overexpression.

To evaluate the functionality of recombinant PDE3B, activation studies were conducted in AdPDE3B-infected INS-1 (832/13) cells. Infected INS-1 (832/13) cells were stimulated with 16 mM glucose, 100 nM insulin or 60 mM K^+^ and PDE3 activity was measured in homogenates. The stimuli resulted in activation of PDE3B although to a lesser degree than in experiments including endogenous PDE3B (Fig. 2D). Nevertheless, this indicates that the recombinant enzyme is responding properly to stimuli in infected cells.

**Figure 3:**
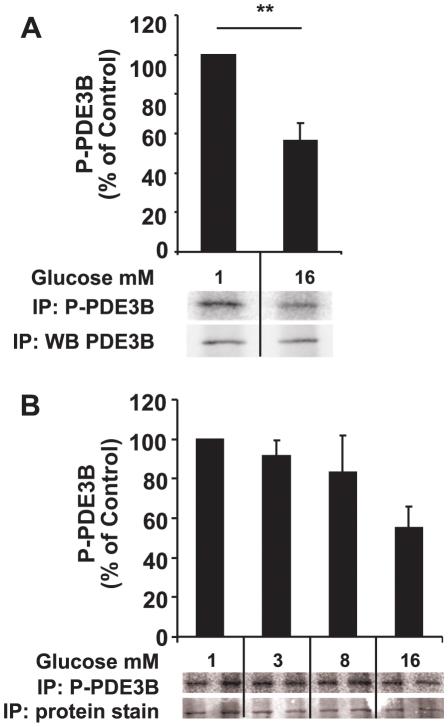
Phosphorylation of PDE3B in response to stimulation with glucose in INS-1 (832/13) cells. Recombinant ^32^P-labelled PDE3B was immunoprecipitated and solubilized from crude membranes, run on SDS-PAGE, subjected to autoradiography and immunoblot analysis (one representative experiment shown). A. Stimulation with 16 mM glucose for 1 hour (n = 5). B. Stimulation with increasing glucose concentrations for 1 h. Quantification was made using a Fuji LAS 3000 Plus system.

**Figure 4:**
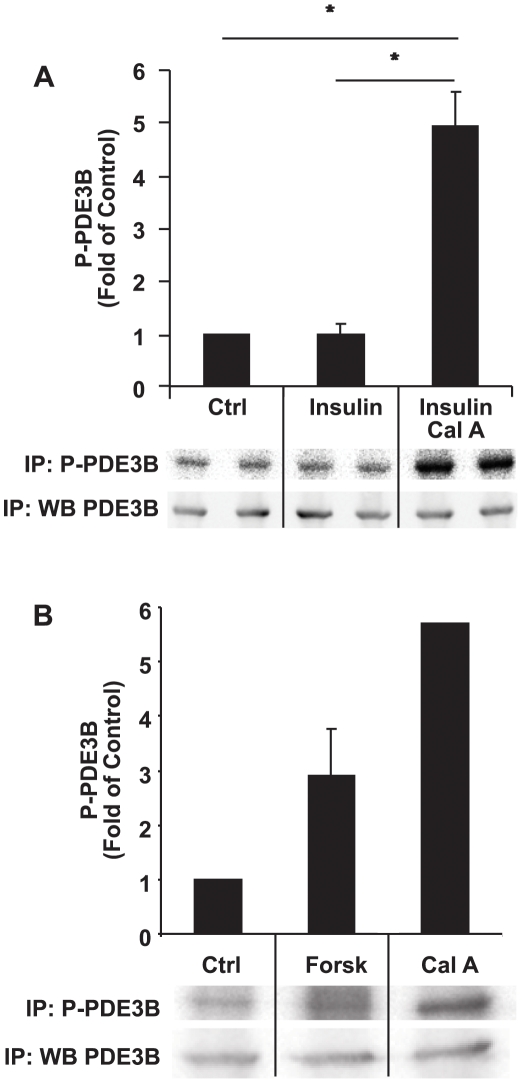
Phosphorylation of PDE3B in response to insulin, forskolin and calyculin A. Immunoprecipitated recombinant ^32^P-labelled PDE3B from crude membranes were run on SDS-PAGE, subjected to autoradiography and immunoblotting (one representative experiment shown). Quantification was made using Fuji LAS 3000 Plus system (n = 4 for A and n = 2 for B).

To study phosphorylation of PDE3B, AdPDE3B-infected INS-1 (832/13) cells were ^32^P-labelled and treated with various agents as indicated in Fig. 3. PDE3B was immunoprecipitated and subjected to SDS-PAGE, immunoblot analysis and autoradiography. Stimulation with 16 mM glucose for 1 hour resulted in a significant, 45% reduction in the phosphorylation of PDE3B (Fig. 3A), in a dose-dependent manner (Fig. 3B). However, stimulation with 100 nM insulin did not result in any apparent change in the amount of phosphorylation of PDE3B (Fig. 4A). On the other hand, the phosphatase inhibitor calyculin A (100 nM) induced a marked increase in the phosphorylation of PDE3B. Also, a marked increase in the phosphorylation of PDE3B was seen when the cAMP-elevating agent forskolin (100 nM) (Fig. 4B) was included, as compared to non-stimulated cells.

### 3.3 PDEs in human pancreatic islets

**Figure 5:**
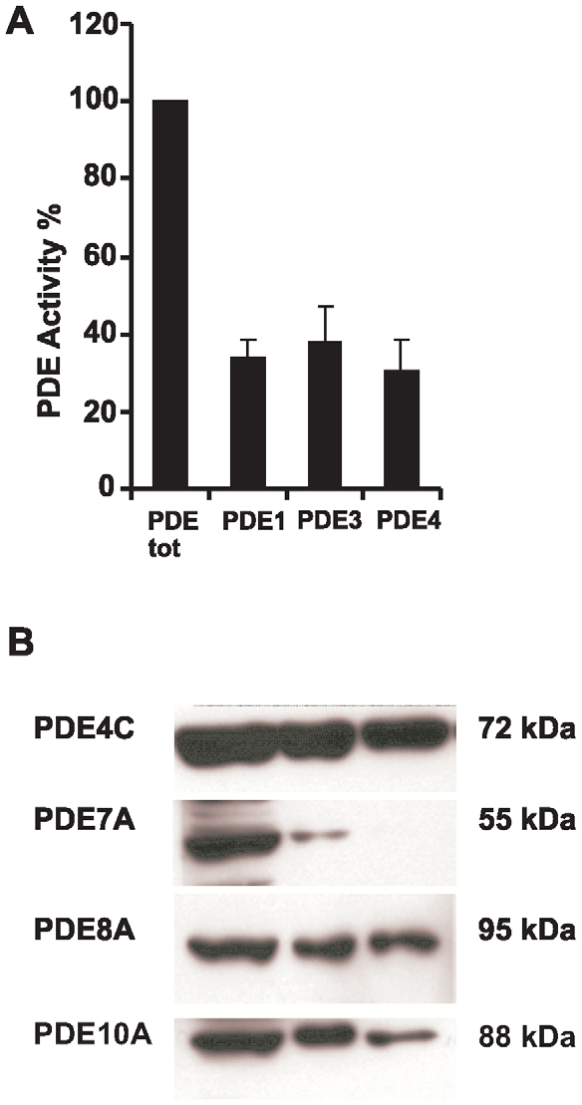
Activity and expression of PDEs in human pancreatic islets. A. Human islets were homogenized and total PDE, PDE1, PDE3 and PDE4 activity was measured (n = 3). B. 20–25 µg of islet homogenates from three different human donors were run on SDS-PAGE and transferred to nitrocellulose membranes. Membranes were subjected to immunoblotting using PDE-isoform selective antibodies (one representative experiment shown).

The enzymatic activity and expression of selected PDEs were studied in human pancreatic islets. Isolated human islets were homogenized and enzymatic activity of PDE1, PDE3, and PDE4 was measured. As shown in Fig. 5A, PDE1, PDE3, and PDE4 each contribute with ∼30–37% of the total PDE activity in human islets.

To identify the expression of selected PDEs, human islets were homogenized and proteins were subjected to immunoblot analysis using antibodies against PDE4C, PDE7A, PDE8A and PDE10A. Fig. 5B shows the expression of PDE isoforms in islets from three human donors identified by immunoblot analysis. As shown in Fig. 5B, we could detect PDE4C, PDE7A, PDE8A and PDE10A. Taken together, these results show that PDE1, PDE3, PDE4C, PDE7A, PDE8A and PDE10A are present in human pancreatic islets.

## Discussion

We have studied the regulation of the cAMP-degrading enzyme PDE3B in rodent pancreatic islets and insulin secreting cells. PDE3B was found to be activated in response to stimuli that are relevant for pancreatic β-cell function and increased PDE3B activity was associated with changes in the state of phosphorylation of the enzyme. In addition, the expression of a number of PDEs in human pancreatic islets was investigated. Thus, in human pancreatic islets activities of PDE1, PDE3 and PDE4 and expression of PDE4C, PDE7A, PDE8A and PDE10A proteins were demonstrated.

PDE3B is expressed in adipose tissue, liver, pancreatic β-cells, and hypothalamus and has been found to regulate many metabolic events central to whole body energy homeostasis [27], [28]. In particular, PDE3B regulates lipid and glucose metabolism in adipocytes [29], [30] and hepatocytes [31], insulin secretion in pancreatic β-cells [11], [15] as well as leptin action in hypothalamus and pancreatic β-cells [28], [32]. In adipocytes, insulin-mediated activation of PDE3B has a key role in antagonizing cAMP-mediated lipolysis [33], [34] and in hepatocytes, insulin-mediated activation of the enzyme is believed to contribute to antagonization of cAMP-mediated glycogenolysis [35]. Thus, the finding that insulin induces activation of PDE3B in β-cell is in agreement with PDE3B as a target for insulin action also in this cell type. With regard to the role of PDE3B activation in response to insulin, it has been shown that leptin and IGF-1 attenuate insulin secretion in a PDE3B-dependent manner in HIT-T15 cells [6], [36]. In the present work, glucose stimulation of rodent islets and β-cells was found to activate PDE3B. Although glucose is the major trigger of insulin release and exogenously added insulin was shown to activate PDE3B in pancreatic islets and β-cells, glucose-stimulated activation of PDE3B is most likely not mediated by an insulin-dependent mechanism. This is based on the finding that diazoxide did not inhibit glucose-stimulated activation of PDE3B.

The finding that high K^+^ (elevated K^+^ concentration depolarizes the β-cell and triggers Ca^2+^ influx and insulin secretion, thus bypassing glucose metabolism) induces activation of PDE3B suggests that glucose-induced activation of PDE3B may be mediated downstream of the metabolization of the sugar, maybe at the level of Ca^2+^ influx. It is known that increased Ca^2+^ gives rise to increased cAMP levels via activation of Ca^2+^-dependent adenylyl cyclases [20], [37]. Thus, it is possible that glucose mediates its effect on PDE3B via a cAMP-dependent mechanism [38]. This is compatible with the finding that the cAMP-elevating agent forskolin induced activation of PDE3B and also in agreement with previous results demonstrating activation of PDE3B in response to cAMP-increasing hormones in adipocytes and hepatocytes [33], [34].

The consequence of activation of PDE3B is expected to be a faster turnover of cAMP and essentially an attenuation of insulin secretion as cAMP has an established role as a potentiator of insulin secretion through PKA-dependent and PKA-independent mechanisms. Thus, we hypothesize that PDE3B activation by glucose, insulin and cAMP-elevating agents constitute a feedback loop to constrain and direct cAMP signals. These results are in agreement with previous studies by us showing that chronic overexpression of PDE3B in β-cells in mice results in reduced insulin secretion [7], [39].

Activation coupled to phosphorylation of PDE3B has been extensively studied in adipocytes [36], [40], [41] and partly in hepatocytes [42] but there are no previous reports concerning phosphorylation of PDE3B in pancreatic β-cells. To be able to study PDE3B activation and phosphorylation in β-cells we used an adenovirus-mediated expression system to overexpress PDE3B. Notably, control experiments with recombinant PDE3B in INS-1 (832/13) cells showed that it could be activated and was localized to the same intracellular compartments as is endogenous PDE3B. Further, we have previously shown that recombinant PDE3B attenuates glucose-induced insulin secretion and GLP-1-potentiated insulin secretion [8], [11].

In agreement with results from adipocytes and hepatocytes, forskolin-induced activation of PDE3B was coupled to an increased total phosphorylation of PDE3B. However, glucose-stimulated activation of PDE3B was coupled to a decrease in total phosphorylation of PDE3B. This is the first time that an increase in PDE3B activity has been coupled to a decrease in total phosphorylation of the enzyme, which could be explained by a glucose-induced activation of a phosphatase dephosphorylating an “inhibitory” phosphorylation site in PDE3B. PP1 and PP2A (ser/thr phosphatases) are the primary phosphatases found in insulin-secreting cells [43], [44]. Indeed, it was recently shown that glucose itself or glucose metabolites can inhibit [45] as well as enhance [46] protein phosphatase activities in insulin-secreting cells. To this end, we have not been able to identify a glucose-stimulated phosphatase acting upon PDE3B, leading to activation of the enzyme. One challenge is the fact that the specificity of phosphatases is determined by a number of different regulatory subunits localized to different locations in the cell.

Stimulation with calyculin A, a phosphatase inhibitor, resulted in a 6-fold increase in PDE3B phosphorylation. However, hitherto we have not been able to detect changes in PDE3B phosphorylation as a result of insulin stimulation. To detect insulin-induced alterations in phosphorylation, it may be necessary to study changes in the phosphorylation of unique phosphorylation sites in PDE3B. In adipocytes, both protein kinase B (PKB) and protein kinase A (PKA) have been suggested kinases for PDE3B [25], [36], [41], [47] and several phosphorylation sites have been identified and shown to be specific for activation by, for example, insulin and isopreterenol [40], [42]. Thus, as it has not been established which kinases phosphorylate PDE3B in β-cells, we suggest that forskolin and insulin as well as calyculin A induce phosphorylation of PDE3B, presumably by activating PKA and PKB, respectively.

Several PDEs were detected in human pancreatic islets including PDE1, PDE3, PDE4C, PDE7A, PDE8A and PDE10A. PDE1, PDE3, and PDE4 activities were estimated using family-selective PDE inhibitors in enzyme assays, whereas the other PDEs were detected using immunoblot analysis. With regard to PDE4, only PDE4C (not PDE4A, B and D) was detected, indicating that PDE4C is the major isoform in human islets. Previously, one single study has shown the presence of PDE3 and PDE4 activities as well as modest activity of PDE1 in human islets [9] and, importantly, that PDE3 inhibition resulted in increased insulin secretion.

With regard to PDEs as potential targets for modulating insulin secretion, a recent study showed that inhibitors selective for PDE10A acted as insulin secretagogues [14] in rat pancreatic islets. Another study stated that siRNA-mediated silencing of PDE8B enhanced GSIS, whereas inhibition of PDE10A did not have a significant effect [13]. Inhibition or knocking down of PDE3B has been shown to be beneficial at the level of the β-cells, leading to improved insulin secretion [6], [48]. However, other metabolic effects of PDE3 inhibition have been associated with overall non-beneficial metabolic phenotypes in mice and rats [49], [50]. It thus seems promising that inhibition of PDEs other than PDE3 potentiates an effect of insulin secretion and can therefore be more advantageous for the treatment of diabetes.

In summary, we conclude that PDE3B, a PDE isoform with important functions in β-cells [7], [8], [11], [15], is activated in response to stimuli relevant for pancreatic β-cell function in rat islet and INS-1 (832/13) cells and that activation is important in feedback modulation/fine tuning of cAMP levels. In addition, increased PDE3B activity is associated with increased as well as decreased phosphorylation of the enzyme. We also conclude that PDE1, PDE3, PDE4C, PDE7A, PDE8A and PDE10A are present in human pancreatic islets.
